# The Gasul Phenomenon Still Alive in the Developing World: A Case Report

**DOI:** 10.4314/ejhs.v33i2.25

**Published:** 2023-03

**Authors:** Muluken Ahmed, Mohammed Nasir, Shibikom Tmrat

**Affiliations:** 1 Pediatrician, Arbaminch University, School of Medicine, Department of Pediatrics, Arbaminch, Ethiopia; 2 Pediatrcs Cardiology fellow, Hawassa university, School of medicine, Department of Pediatrics, Hawassa, Ethiopia; 3 Pediatics and Adult Cardiac Surgeon, Saint Paul's Hospital Millennium Medical College, Department of Surgery, Addis Ababa, Ethiopia

**Keywords:** Patent ductus arteriosus, peri membranous ventricular septal defect, right ventricular outflow tract obstruction, Gasul phenomena, Case report

## Abstract

**Background:**

In child, ventricular septal defect is the most prevalent congenital cardiac disease. Some ventricular septal defects have the potential for spontaneous closure. In poor nations closure based on indications may not be feasible. The patient's natural course may therefore be observed. The Gasul phenomenon, a right ventricular outflow obstruction, is one of the complications.

**Case Presentation:**

A 7-year-old child who had recurrent pneumonia, poor weight gain, and excessive sweating eventually had these symptoms go away. A large peri membranous ventricular septal defect and a small patent ductus arteriosus was detected on echocardiography during infancy. Later, the patient acquired a muscular ridge across the right ventricular outflow tract. Muscular ridge excision and closure of patent ductus arteriosus and ventricular septal defect were done. Patient was discharged in stable condition.

**Conclusion:**

Right ventricular outflow tract blockage can be avoided by performing early surgical closure of a ventricular septal defect.

## Introduction

By definition, any opening in the ventricular septum is known as a ventricular septal defect. It is the most prevalent congenital heart disease in children, occurring in 50% of cases in combination with other congenital heart diseases and 20% in isolation. Despite the possibility of spontaneous closure, certain types of ventricular septal defects never close at all (1).

Even if there is a clear indication for surgery, many patients in underdeveloped nations may be unable to undergo surgery due to financial constraints, allowing the natural course of the disease to be determined (2). one of the natural histories of un-operated ventricular septal defect was Gasul phenomenon which was described by Dr. Gasul seven decades back in an autopsy of the patients with a large ventricular septal defect which transformed into progressive infundibular narrowing through time (3).

The purpose of this case report is to describe the presentation, diagnosis, and management of a 7-year-old male child who developed infundibular stenosis during follow-up for large perimembranous ventricular septal defect partially restricted by the septal leaflet of the tricuspid valve.

## Case Presentation

A 7-year-old male child presented for the first time in the cardiac center of Ethiopia at the age of 6 months with a history of excessive sweating during breastfeeding with interruption of breastfeeding. He also had a history of poor weight gain and frequent pneumonia till he was three years old. After 3 years of age, there has been no incidence of pneumonia, and the child has begun to grow well. Physical examination at presentation showed a grade 3 holosystolic murmur in the left lower sternal border later he developed an ejection systolic murmur on the same area with the splitting of the second heart sound.

**Figure 1:**
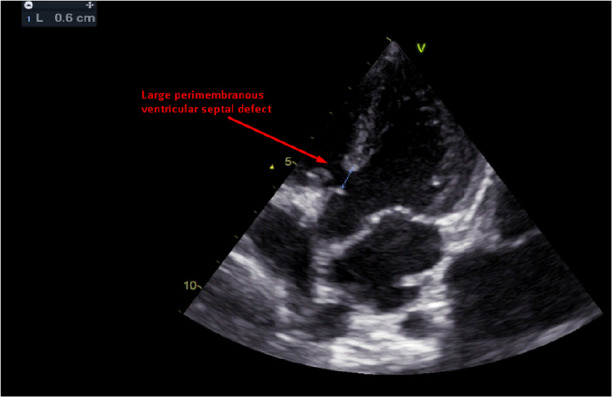
Large peri-membranous ventricular septal defect at age of 7 months

The ECG findings at 7 months of age were sinus rhythm, prominent left ventricular forces, left ventricular dilatation, and normal axis deviation. A chest x-ray was done at the same age and showed mild cardiomegaly. Additionally, the echocardiographic finding was large perimembranous ventricular septal defect 7 mm, left to right shunt, and partially restricted by septal leaflet of tricuspid valve with significant trans- ventricular septal defect gradient (Figure 1).

**Figure 2:**
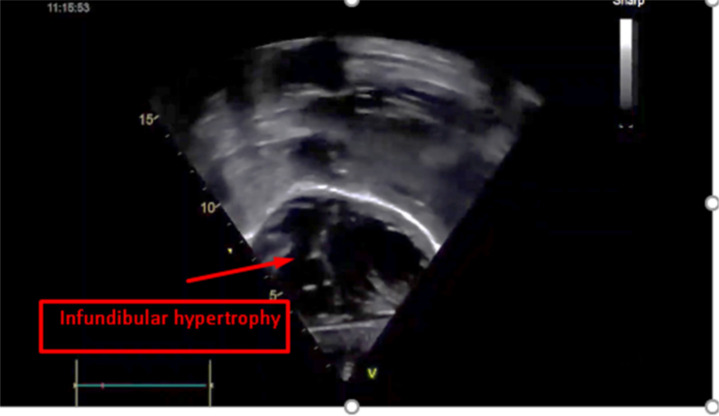
Development of muscular ridge over the right ventricular outflow tract at age of 6 yrs and 7 months

**Figure 3:**
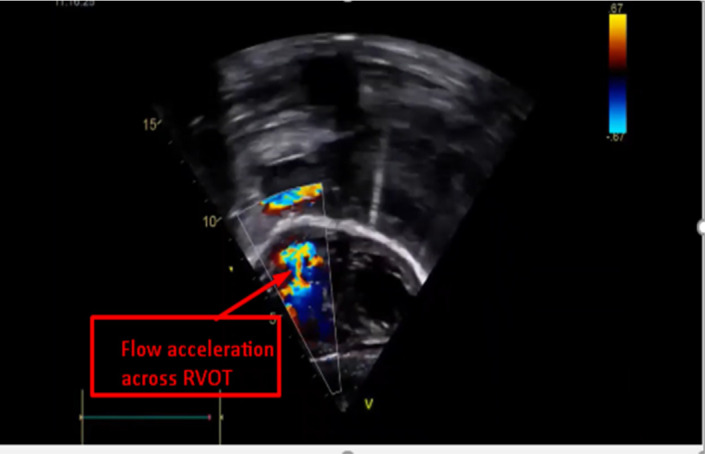
Acceleration across right ventricular outflow tract

There was also a small patent ductus arteriosus, 3mm a left to right shunt. Repeat echocardiography at the age of four demonstrated the decrement of the perimembranous ventricular septal defect. The last echocardiography before the surgery at the age of 6 years and 7 months showed obstructive muscular ridges over the right ventricular outflow tact with flow acceleration (Figure 2 and 3). In addition, the ventricular septal defect closed spontaneously with no residual.

Patent ductus arteriosus ligation with fibromuscular ridge resection from the right ventricular outflow tract was done. Postoperative echocardiography showed no residual patent ductus arteriosus and laminar flow across the right ventricular outflow tract. The patient was discharged home on the third day postoperatively. He has regularly followed up in the cardiac center of Ethiopia.

## Discussion

Dr. Gasul observed a clinical improvement in congestion symptoms in children with large ventricular septal defects after a year of life. He speculated that infundibular stenosis, which reduces pulmonary blood flow, could be the origin of this occurrence, and the disease was later labelled the Gasul phenomenon (3). Hypertrophy of the crista supraventricularis, distortion of the parietal and septal bands, and deformity with infundibulum stenosis may explain the natural history of ventricular septal defect in this phenomenon (4).

Because of the well-developed pulmonary artery and right ventricular outflow tract, surgical correction of this acquired infundibular stenosis is easier than tetralogy of Fallot (5). However, because Ethiopia has only one cardiac center for the entire country, operation may not be performed as soon as possible.

In conclusion, closure of ventricular septal defect based on guideline recommendations has paramount importance in preventing complications like right ventricular outflow tract obstruction that develops due to formation of muscular ridge.
